# Risky Business: Predator Chemical Cues Mediate Morphological Changes in Freshwater Snails

**DOI:** 10.1093/iob/obae033

**Published:** 2024-09-02

**Authors:** M J Wagner, P A Moore

**Affiliations:** Laboratory for Sensory Ecology, Department of Biological Sciences, Bowling Green State University, Life Science Building 217, Bowling Green, OH 43403, USA; University of Michigan Biological Station, 9133 Biological Road, Pellston, MI 49769, USA; J.P. Scott Center for Neuroscience, Mind, and Behavior, Bowling Green State University, Psychology Building 206, Bowling Green, OH 43403, USA; Laboratory for Sensory Ecology, Department of Biological Sciences, Bowling Green State University, Life Science Building 217, Bowling Green, OH 43403, USA; University of Michigan Biological Station, 9133 Biological Road, Pellston, MI 49769, USA; J.P. Scott Center for Neuroscience, Mind, and Behavior, Bowling Green State University, Psychology Building 206, Bowling Green, OH 43403, USA

## Abstract

Many prey organisms respond to the nonconsumptive effects of predators by altering their physiology, morphology, and behavior. These inducible defenses can create refuges for prey by decreasing the likelihood of consumption by predators. Some prey, as in marine mollusks, have been shown to alter their morphology in response to the presence of size-limited predation. To extend this work, we exposed pointed campeloma snails (*Campeloma decisum*) to chemical cues from a natural predator, the rusty crayfish (*Faxonius rusticus*), to better understand how snail morphology changes under the threat of predation. The total force needed to crush shells, total shell length, aperture width, and total weight, along with changes to these 3 body measurements, were recorded for each individual and used to quantify morphological changes as a function of risk. Snails exposed to crayfish chemical cues had shells that required significantly more force to crush their shells than controls (*P* = 0.023). Total shell length was greater in crayfish-exposed snails than in control snails (*P* = 0.012), and snails in the crayfish treatment also showed significantly more change in shell length than control snails (*P* = 0.007). Similarly, aperture width was significantly greater in exposed snails (*P* = 0.011). However, exposed snails exhibited significantly less change in aperture width than controls (*P* = 0.03). Finally, we found that snails exposed to crayfish weighed significantly more than snails in the control (*P* = 0.008). Thus, the results of this study show that morphology of gastropods is altered in the presence of predators, and this may be an antipredator tactic directly related to predation risk.

## Synopsis (Swedish)

Många bytesorganismer reagerar på de icke-konsumerande effekterna av rovdjur genom att förändra deras fysiologi, morfologi och beteende. Dessa inducerbara försvar kan skapa tillflyktsort för bytesdjur genom att minska sannolikheten för konsumtion av rovdjur. Vissa byten, som i marina blötdjur, har visat sig förändra sin morfologi som svar på förekomsten av storleksbegränsad predation. För att utöka detta arbete, exponerade vi spetsiga campelomasniglar (*Campeloma decisum*) för kemiska signaler från ett naturligt rovdjur, den rostiga kräftan (*Faxonius rusticus*), för att bättre förstå hur snigelmorfologi förändras under hot om predation. Den totala kraften som behövs för att krossa skal, total skallängd, öppningsbredd och totalvikt, tillsammans med ändringar av dessa tre kroppsmått, registrerades för varje individ och användes för att kvantifiera morfologiska förändringar som en funktion av risk. Sniglar som exponerats för kemiska signaler från kräftor hade skal som krävde betydligt mer kraft för att krossa sina skal än kontroller (*P* = 0,023). Den totala skallängden var större hos kräftexponerade sniglar än kontrollsniglar (*P* = 0,012), och sniglar i kräftbehandlingen visade också signifikant mer förändring i skallängd än kontrollsniglar (*P* = 0,007). På liknande sätt var bländarbredden signifikant större hos exponerade sniglar (*P* = 0,011). Men exponerade sniglar uppvisade signifikant mindre förändring i öppningsbredd än kontroller (*P* = 0,03). Slutligen fann vi att sniglar exponerade för kräftor vägde betydligt mer än sniglar i kontrollen (*P* = 0,008). Således visar resultaten av denna studie att morfologin hos gastropoder förändras i närvaro av rovdjur, och detta kan vara en antipredatortaktik som är direkt relaterad till predationsrisk.

## Introduction

Predators may affect prey in two very broad ways: through direct consumption of prey (lethal effects) or by altering prey response to predators in some way (nonlethal effects such as changes in foraging behavior or behavioral time budgets) (Lima and Dill 1990). Previous studies have shown that nonlethal effects tend to have a larger influence upon prey ecology compared to lethal effects (Preisser et al. 2005; Matassa and Trussell 2011). Because of this, the responses of prey to predators depend heavily on the ability of prey to assess the risk that predators impose.

A prey's perception of risk is extracted from predatory cues, and from this information, prey can take actions that alleviate the risk associated with various activities such as foraging or mating when vigilance is typically low (Zöttl et al. 2013). Though many prey species alter behavior in the presence of predators, other changes also may occur as a function of predator presence. Some prey organisms exhibit changes to body size in riskier situations, as larger body size may make it harder for predators to successfully capture and consume prey (Januszkiewicz and Robinson 2007). When given a choice, xanthid crabs (*Micropanope* sp.) killed significantly fewer queen conchs (*Strombus gigas*) that had larger bodies compared to queen conch individuals with smaller overall body sizes, showing that xanthid crabs preferred relatively smaller individuals (Ray-Culp et al. 1999). In this way, larger body sizes likely acted as a refuge for queen conchs from xanthid crab predators (Ray-Culp et al. 1999). Observed variation in such changes offers evidence to the well-documented idea that antipredator tactics are plastic and may be altered by the magnitude of risk to which prey are exposed (Orizaola et al. 2012).

Predator-induced defenses in the form of morphological changes may ultimately reduce the predation risk experienced by prey (Chivers et al. 2008). Three-spined sticklebacks (*Gasterosteus aculeatus*) have been known to exhibit morphological plasticity and grow larger spines and deeper bodies when exposed only to predator odors (Frommen et al. 2011). However, many predators may be limited in the size of prey they can consume, and this size limitation changes with growth in the prey and predator. Gape limitations are found in some fish and reptile predators, meaning that these predators are unable to swallow prey that are larger than their gape (Hill et al. 2004). Predation risk, in these systems, is based on the relative size of prey and the predatory mechanism (mouth, claw, etc.). Changes in prey morphology or body shape could reduce that risk (Brönmark et al. 1999). Similar to gape limitation, other predators may be size limited due to chelae size. Some crustacean predators (including crab, lobster, and crayfish species) catch and break apart prey utilizing large, claw-like appendages (chelae) (Boßelmann et al. 2007). However, these predators are limited to catching prey that are able to fit within chelae, as handling larger prey items is disadvantageous for the predator and offers prey an easier means to escape (Juanes 1992). Larger crustaceans that have larger chelae also typically have stronger pinch forces (Benson and Clotfelter 2023). Size limitations become very valuable from a prey's perspective because changes to morphology may actually create a refuge for prey to evade capture. Because of these potential refuges, understanding the mechanisms that may cause changes in prey morphology leads to a more complete picture of predator–prey ecology.

Though many studies within the framework of predator–prey ecology have worked to understand the numerous effects predators have on prey (see Sih et al. 1985; Apfelbach et al. 2005; Sheriff et al. 2020), there is still a need to examine the various morphological changes that prey may exhibit in risky habitats. Crayfish are a common organism found throughout North America and tend to be omnivorous, feeding on plant matter and detritus, as well as acting as a predator on other benthic species. Because crayfish affect organisms on multiple trophic levels and have been known to alter ecosystem processes, they are also described as keystone species (Reynolds et al. 2013). Finally, crayfish also tend to be size-limited predators, as they are constrained to consuming organisms that can be crushed within their chelae. Thus, we chose to utilize one species of crayfish, the rusty crayfish (*Faxonius rusticus*), as predators for this project. Because crayfish are generalists, they tend to forage on a variety of plants and benthic organisms, including the pointed campeloma snail (*Campeloma decisum*). The pointed campeloma snail is important for lentic and lotic food webs, as various species prey upon these snails (Van Appledorn et al. 2007). This species also tends to have a relatively simple shell shape when compared to other viviparid snail species (Jokinen 1984). The pointed campeloma also tends to filter feed and consume organic material found on the substrate on aquatic habitats (North and Minton 2021). Finally, gastropods, including the pointed campeloma, utilize shells as a morphological antipredator defense to prevent predators from damaging the soft tissue located within the shell (Vermeij 1978). Many gastropods have been documented as developing stronger shells in response to predation threat, either by altering thickness or by altering microstructure of the shell (Brandwood 1985; Trussell and Nicklin 2002; Avery and Etter 2006).

In line with this thinking, we designed an experiment to test the nonconsumptive effects of crayfish on pointed campeloma morphology. We were particularly interested in how predator exposure altered the strength of snails’ shells as well as the overall body size of snails, as these changes could illustrate a change in antipredator tactics and inducible defenses that provide refuge against crayfish predators. We hypothesized that snails exposed to crayfish would have stronger shells as well as larger overall body sizes when compared to snails that did not receive predator cues.

## Methods

### Experimental design

We designed an experiment that allowed us to measure changes in snail growth (length, aperture width, and weight) and shell strength when snails were exposed to predatory chemical cues from crayfish. Changes to overall growth and shell strength were compared for snails in control and crayfish treatments in order to appropriately ascertain the role of predator presence in snail morphology and predator defense. The crayfish treatment contained one rusty crayfish, while control trials had no crayfish present. In trials containing crayfish, each crayfish was attached to a tether in order to test the nonconsumptive effects of predators onto snail prey, and all snails were contained within a mesh bag to aid in ease of retrieval at the end of trials and to protect the snails from predation. Each snail had initial and final measurements recorded in order to calculate changes to growth. All trials in the crayfish treatment utilized the same crayfish from the beginning to the end of the trial. If a crayfish escaped, molted, or died, an immediate replacement crayfish was added to the respective mesocosm. Replacement crayfish were not size matched as the only role of crayfish for this experiment was to be an odor donor. A total of 32 trials were run: 16 control trials without crayfish and 16 trials with one crayfish. Each trial had a total of 12 snails contained in three bags with four snails per bag. A total of 384 snails were used in this experiment. Of this total, one snail died before the end of the trial and the final measurements could not be used.

In addition to the mesocosm trials, 47 snails were caught before the last sampling day and measured to provide comparative data from natural specimens (Supplemental Data). These 47 snails (total shell length 1.84 ± 0.068 cm [mean ±  SEM]; aperture width = 1.03 ± 0.035 cm [mean ± SEM]; and weight = 1.47 ± 0.12 g [mean ± SEM]) were considered natural and their data placed in the Supplemental Data for some context.

### Collection and housing of organisms

#### Crayfish

Two-hundred male rusty crayfish (*F. rusticus*) (postorbital carapace length = 2.47 ± 0.07 cm [mean ± SEM]) were captured in minnow traps baited with sardines from Carp Lake River in Emmet County, Michigan, USA (45.7497°N, 84.8292°W). Because crayfish only served as odor donors for this project, form I and form II (reproductive and nonreproductive, respectively) males were used, as well as males that did not have all appendages fully intact. All crayfish were kept in a flow-through steel cattle trough (200 cm × 60 cm × 60 cm [length × width × depth]) with unfiltered river water from the East Branch of the Maple River (45.5280°N, 84.7738°W) fed through PVC pipes. Crayfish were able to feed on naturally occurring detritus contained within the river water. In order to increase the aversiveness of crayfish chemical cues for snails, crayfish were also fed crushed snails three times over the course of the experiment, though these feedings only took place in the stock tank that crayfish were held in and not within experimental mesocosms, as we did not want snails to respond to conspecific alarm cues. Crayfish were housed outside under the natural daylight:darkness (15.5 h:8.5 h) regime and natural temperatures. Upon completion of the project, crayfish were not returned to the river due to the nonnative status of this species and were frozen according to collection permit requirements.

#### Pointed campeloma

Campeloma snails (*C. decisum*) (initial shell length = 2.10 ± 0.02 cm [mean ± SEM]; initial aperture length = 1.14 ± 0.01 cm [mean ± SEM]; and initial weight = 2.04 ± 0.06 g [mean ± SEM]) were collected from Douglas Lake in Cheboygan County, Michigan (45.5770°N, 84.6929°W) prior to the start of the experiment. We collected approximately 400 snails by kicking up sediment along the lakeshore to uncover buried snails. Because we wanted a range of sizes for this experiment, all snails that were found were collected and placed into a bucket before being carried back to the University of Michigan Biological Station Stream Research Facility. Once at the lab, snails were placed in a 10-gal aquarium (50.8 cm × 25.4 cm × 30.5 cm [length × width × depth]) that was filled with unfiltered river water from the East Branch of the Maple River. The tank had a sand substrate layer (∼6 cm depth) to allow snails to burrow and was aerated. Water was also changed weekly as water replacement ensured that evaporation was kept to a minimum and provided more detritus for snails to feed upon. Water in both the mesocosm and the snail tanks was drawn from the Maple River, which, like most lakes and rivers in this section of Michigan, resides over a limestone bedrock. CaCO_3_ levels typically run 175 mg/L or higher and should not be a limiting nutrient in the shell productive of the snails. After the project was completed, snails were brought back to the Laboratory for Sensory Ecology at Bowling Green State University in Bowling Green, Ohio.

### Experimental mesocosms

Thirty-two flow-through mesocosms (60.9 cm × 40.6 cm × 20.3 cm [length × width × depth]) were constructed from cinderblocks lined with 4-mil (0.004-cm-thickness) polyethylene sheeting. Four 208-L plastic drums served as constant head tanks for mesocosms, as one head tank was able to supply unfiltered water from the East Branch of the Maple River to eight mesocosms. Water entered the drums via a PVC pipe that was covered in nylon stockings to prevent excess detritus and/or macroinvertebrates entering the mesocosms. Water was delivered from the drums to each mesocosm using a garden hose. Black corrugated pipe was filled with sand and placed horizontally at the downstream end of each mesocosm, and water was able to flow over the pipe to exit the stream. The plastic sheeting was draped over top of the end pipe. This kept water depth at approximately 10 cm. Each mesocosm was lined with sand substrate. Throughout the trial period, each mesocosm accumulated detrital material on the substrate as well as algal growth on the sides of the mesocosm. Crayfish fed on these materials for the length of the experiment. The upstream end of the mesocosm contained a crayfish and crayfish tether, while the downstream end contained three mesh containers, each holding four snails. The bags were placed parallel to the corrugated pipe in a random order. Because all mesocosms were outside, the daily air and water temperature as well as sunlight exposure remained relatively similar for each mesocosm throughout the extent of the experiment. Because each mesocosm was fed with unfiltered river water and the bags were placed in full sunlight, both detritus and algal growth were seen in and on the bags providing snails with food during the experiment.

### Experimental protocol

All trials began on May 31, 2023, and ended on either August 1, 2023, or August 2, 2023 (a total of 63 or 64 days, respectively). Differences in end times were due to long data collection days that were divided in half. On the first day of setup, each crayfish was attached to a tether to ensure crayfish did not escape the mesocosm. Tethers were constructed by tying the fishing line around clay tiles and attaching Velcro to the loose end of the fishing line. A second piece of Velcro was attached to each crayfish's carapace with Gorilla Glue^®^ super glue gel. Superglue was allowed to dry for 15 min before placing crayfish into mesocosms so glue had time to set. This system of tethers has been used in the past to keep crayfish within a mesocosm with minimal disturbance to their behavior (Alacantara et al. 2019). Once crayfish were in the mesocosms, 12 snails were randomly selected from the holding tank and three initial measurements were recorded: total length of the shell (from the aperture to the apex of the shell [to the nearest 0.01 cm]), the aperture length (to the nearest 0.01 cm), and the total weight (to the nearest 0.001 g). Snails were divided into three groups of four before being placed into mesh containers (Garton and Johnson 2000). There was no difference between the initial length of snails placed in either the control or crayfish groups (linear mixed model: *F*_(1,30,0.05)_ = 3.31, *P* = 0.08, control snail length 2.1 [± 0.03 SEM] cm, and crayfish snail length 2.2 [± 0.03 SEM] cm). Containers were constructed by hot gluing strips of mesh screening to a Petri dish and tying the top of the mesh with a rubber band. After labeling of bags was finished, bags were placed in a random order parallel to the corrugated pipe. Snails were contained to test the inducible defenses due to the presence of crayfish predators as well as to allow for ease of finding snails on the final day of measurements.

Each day at 08:30, mesocosms were checked. First, researchers ensured crayfish had not escaped, molted, or died overnight. If this happened, a replacement crayfish was immediately measured and added to the stream, and a note was made in the data file. Thirty-five crayfish were replaced in 14 of the 32 stream mesocosms over the course of 10 weeks. Next, crayfish tethers were checked to ensure that the fishing line was intact and not tangled around the water hose or mesh bags, as crayfish were allowed to explore mesocosms. Mesocosms were then hand cleaned to avoid excess detritus accumulating on the endpiece corrugated pipe and slowing water flow. Mesh bags were also hand cleaned to remove excess detritus and algae each day. If mesh bags had moved overnight, they were placed back into a line at the downstream end of the mesocosm. Finally, water hoses were cleaned each day to make sure hoses were not clogged and impeding water flow to mesocosms.

On the final day of the experiment, snails were removed from bags, identified, and had all three measurements retaken so as to compare whether body size and mass had changed. A fourth measurement, crush force, was also taken for each snail. To determine whether shell strength was different across treatment groups, researchers placed snails in a Petri dish and crushed each snail using a spring tension compression tester (INTBUYING© 500N Spring Tension Compression Tester Tensile Testing Machine). Snails were placed on the center of the Petri dish in the same positioning and crushed until an audible cracking sound could be heard. To alleviate bias in the crushing process, the same person crushed all snails and checked to make sure all shells had cracked before discarding the shell and recording the total force (N) needed to break the shell as displayed by the compression tester.

### Data processing

In order to assess changes to shell total length, aperture length, and weight, each snail's initial measures were subtracted from each snail's final measures to produce a change in growth measurement over time. This allowed researchers to see whether growth or a reduction in size had occurred. This process was utilized for all trials. Crush force was also recorded for all snails at the end of a trial. Obviously, crush force can only be obtained at the end of the entire experiment. In order to eliminate bias of force based on size (i.e., larger snails having a higher force simply because of differences in body size), all forces were divided by total body length as well as total body weight. This method has been previously used to standardize crush force data before statistical analysis occurs (Belgrad et al. 2023). This then produced two more variables: crush force per length and crush force per weight. Standardization was utilized across all trials.

Dependent variables consisted of a set of singular final measurements (final total length of snails, aperture width, total weight, crush force, crush force per length, and crush force per weight) and a set of change data (change in total length, change in aperture width, and change in total weight). Data conditioning and treatment followed the steps outlined in Zuur et al. (2009) for mixed-effects models. The first step in this process was creating dot charts to examine potential outliers within the dataset. Four outliers occurred within the 431 snails, so these data were removed before analysis occurred. A collinearity analysis was performed between the dependent variables of total change in shell length, aperture length, weight, and crush force, as well as the variables total length, aperture length, and weight. The total length, shell length, and weight were all highly correlated. However, these variables were never run together in the same model. Next, histograms, q–q plots, and Shapiro–Wilk tests of normality were used to examine the underlying distribution of response variables. All variables had nonnormal distribution. Next, “BestNormalized” was run to determine which data transformation was likely to produce the best normalized dataset (Peterson 2021). Total shell length, aperture length, weight, and crush force per weight were not normally distributed, so a Yeo–Johnson transformation was performed on these datasets. Total crush force and crush force per length were not normally distributed, so a square root transformation was applied to these data. Finally, to normalize total length change, aperture length change, and weight change, order norm was utilized. All variables were normally distributed after this transformation.

### Statistical analysis

An initial statistical analysis of the crushing force as a function of snail size (length) and treatment (crayfish, control, and the naturally caught snails) was performed using an analysis of covariance in R followed by a Tukey-Honest Significant Differences (HSD) analysis. All dependent variables were analyzed using a linear mixed-effects model by running the “lmer” function from the “lmerTest” package in R given the presence of multiple organisms within the same stream (Kuznetsova et al. 2017; R Core Team 2023). Each model was constructed using a single categorical factor (treatment) and two random-effects terms that were nested (snail bag was nested inside of mesocosm). Since there were three bags with four snails in each bag placed in each stream mesocosm, the stream mesocosm was used as the random effect in the mixed models with snail bag nested within mesocosm. The outputs were extracted using the “anova” and “summary” functions from the “car” package in R (Fox and Weisberg 2019). Additional post-hoc analyses were run using the “emmeans” function from the “emmeans” package in R in order to determine whether the treatment produced significant effects (Lenth 2023).

## Results

### Crush force data

**Fig. 1:**
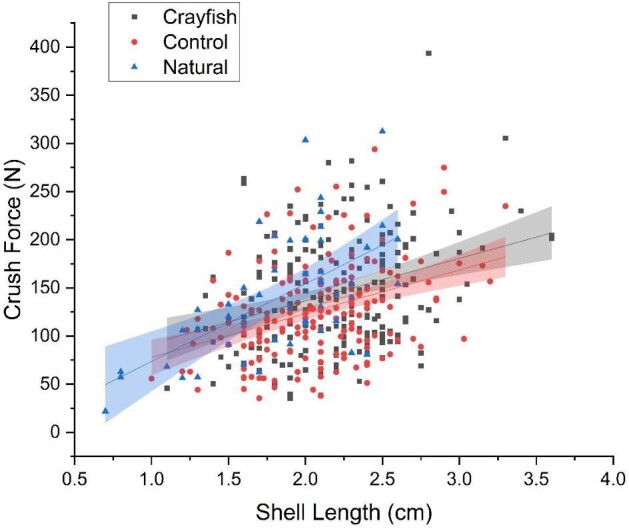
The total crush force of snails as a function of total shell length is shown for three different groups. Crayfish exposed snails are represented by solid squares, control snails are represented by solid circles, and natural snails that were collected and then immediately measured and crushed to serve as a natural baseline are represented by solid triangles. Using a linear mixed model, we found that snail size and treatment significantly influenced the total force needed to break shells (*P* < 0.0001). Upon completion of Tukey-HSD post-hoc analyses, we saw significant differences for crush force, size, and all treatments (*P* < 0.05).

The initial model of crush force as a function of snail size (length) and treatment, which included the naturally caught snails, showed a significant interaction with snail size and treatment influencing the force required to crush the snail shell (*F*_(2,425,0.05)_ = 71.19, *P* < 0.0001). A subsequent post-hoc test showed that the relationship between crush force, size, and the treatments was significantly different for all three comparisons (*P* < 0.05 Tukey-HSD; Fig. 1, Table 1).

**Table 1:** Averages (±SEM) of all measures taken for each dependent variable across all treatments.

Dependent variable	Treatment	Average ± SEM
Total crush force	Control	126.61 ± 3.56
	Crayfish	145.60 ± 4.34
Crush force per length	Control	61.69 ± 1.63
	Crayfish	66.98 ± 1.97
Crush force per weight	Control	80.82 ± 3.31
	Crayfish	77.67 ± 3.44
Total length	Control	2.07 ± 0.03
	Crayfish	2.20 ± 0.03
Change in length	Control	−0.002 ± 0.004
	Crayfish	0.02 ± 0.004
Aperture width	Control	1.16 ± 0.02
	Crayfish	1.23 ± 0.02
Change in aperture width	Control	0.051 ± 0.006
	Crayfish	0.028 ± 0.006
Total weight	Control	1.93 ± 0.08
	Crayfish	2.30 ± 0.09
Change in weight	Control	0.005 ± 0.02
	Crayfish	0.0005 ± 0.01

**Fig. 2:**
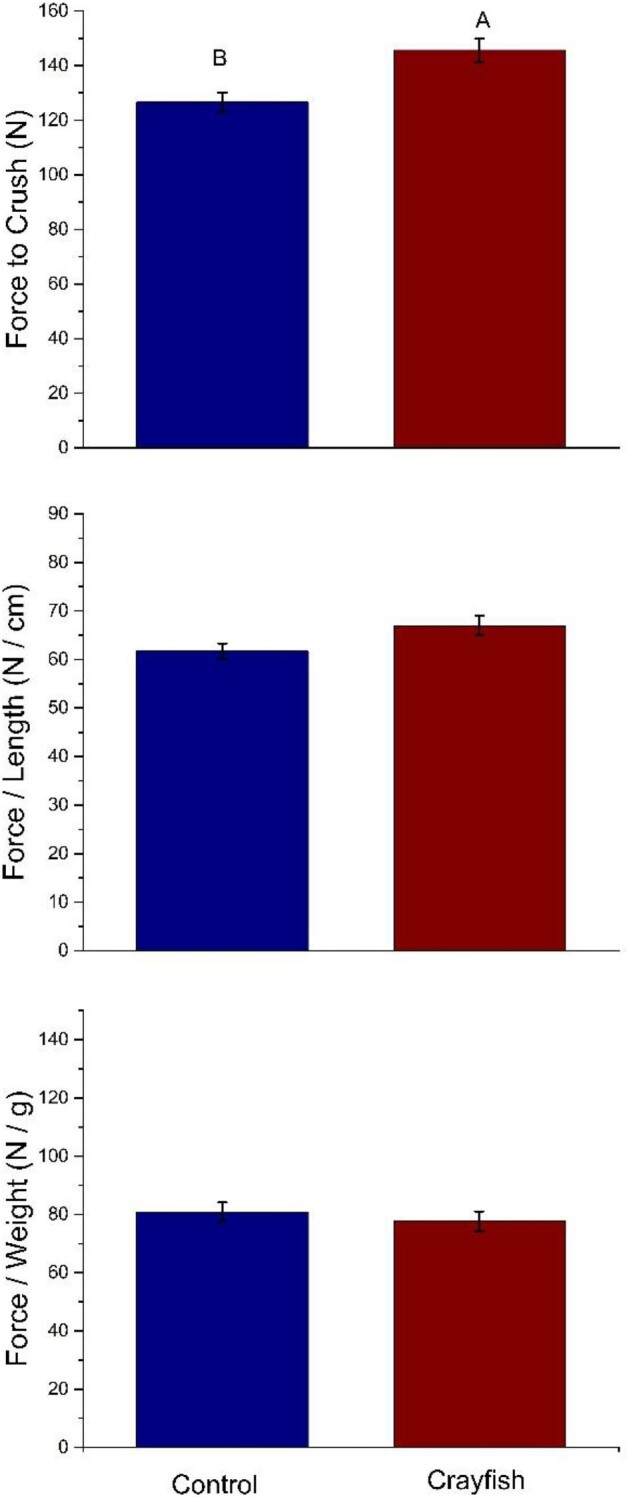
Force (top panel), force per snail length (middle panel) and force per weight (bottom panel) needed to crush a snail within different treatments. The measurement is the mean (±SEM) for snails in the control (left bar) and crayfish (right bar) treatments. Within each panel, bars with different capital letters above the bar indicate a significant difference using an linear mixed model followed by a Tukey-HSD post hoc test. For total force, snails in the crayfish treatment required more force to crush their shell than snails in the control treatment (*P* = 0.023). For force per length, we found no significant differences between treatment groups (*P* = 0.18). There were no significantly different treatment effects observed for crush force per weight (*P* = 0.41).

We analyzed the total force needed to crush snail shells as a function of predator presence to understand the ways in which predator presence affected shell strength. We also analyzed the total force per length and width of each individual. The total force needed to crush snail shells had significant treatment effects between control and crayfish treatments (*F*_[1,30,0.05]_ = 5.7, *P* = 0.023; Fig. 2). Specifically, the total force needed to crush shells was greater in the crayfish treatment than in the control treatment.

Because the force needed to crush a shell could be a function of the total size of the snail, the force per length and force per weight were also analyzed statistically. First, the total crush force needed per snail total length showed no difference between treatments (*F*_[1,30,0.05]_ = 1.88, *P* = 0.18; Fig. 2). Those snails exposed to crayfish needed more force per shell length in order to induce crushing. Finally, crush force per total weight was not significantly different across treatments (*F*_[1,30,0.05]_ = 0.67, *P* = 0.41; Fig. 2).

### Growth data

**Fig. 3:**
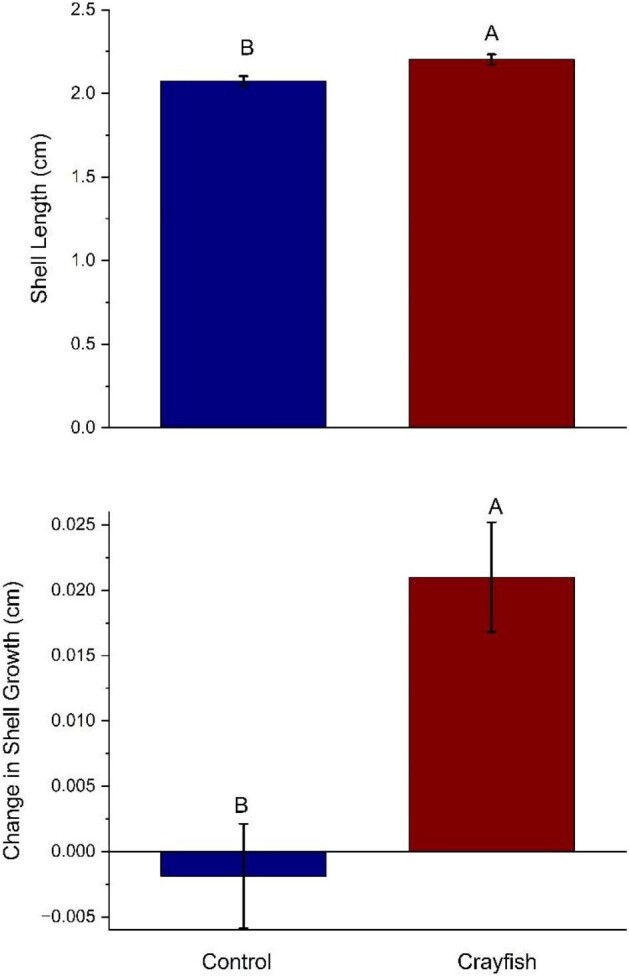
Shell length (top panel) and change in shell growth (bottom panel) within different treatments. The measurement is the mean (±SEM) for snails in the control (left bar) and crayfish (right bar) treatments. Within each panel, bars with different capital letters above the bar indicate a significant difference using an linear mixed model followed by a Tukey-HSD post hoc test. For total length, snails in the crayfish treatment were significantly longer than both the control treatment (*P* = 0.012). For change in shell length, snails in the crayfish treatment exhibited significantly more change than snails in the control treatment (*P* = 0.007).

In order to understand the ways in which overall body size was affected by predator presence, we also calculated three body size measurements (total shell length, aperture width, and total weight), and determined how these measurements changed from the start to the end of the experimental period. First, the total length of snail shells showed significant treatment effects (*F*_[1,30,0.05]_ = 2.64, *P* = 0.012; Fig. 3). When comparing the two treatments, snails in the crayfish treatment had significantly longer shells than controls. We found significant differences in changes in shell length between our two treatment groups (*F*_[1,30,0.05]_ = 9.45, *P* = 0.007; Fig. 3). Specifically, snails exposed to crayfish exhibited more change than those in the control treatment.

**Fig. 4:**
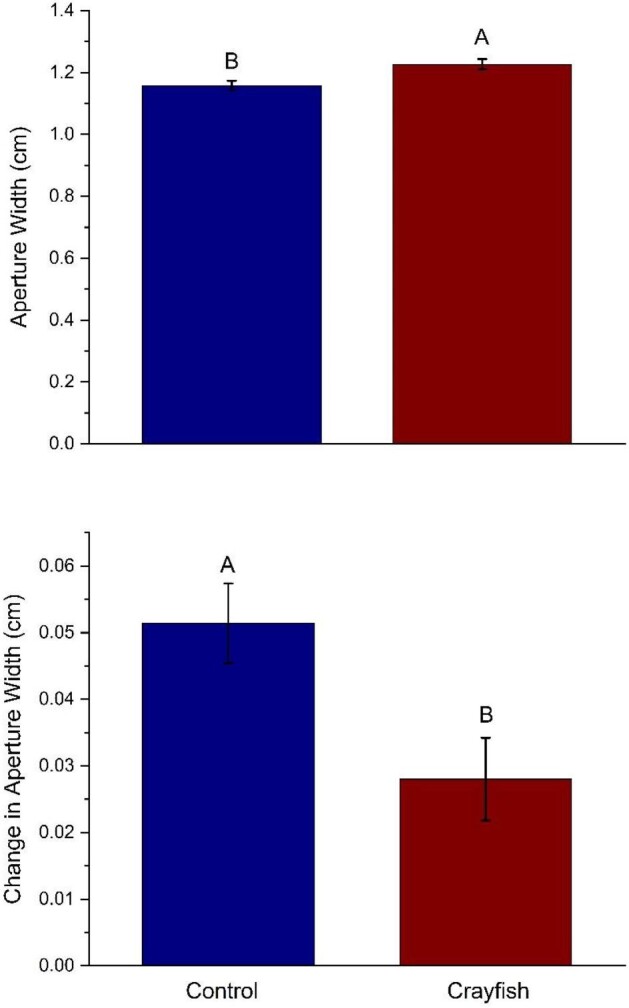
Aperture width (top panel) and change in aperture width (bottom panel) within different treatments. The measurement is the mean (±SEM) for snails in the control (left bar) and crayfish (right bar) treatments. Within each panel, bars with different capital letters above the bar indicate a significant difference using an linear mixed model followed by a Tukey-HSD post hoc test. Snails in the crayfish treatment had significantly wider apertures than those in control trials (*P* = 0.011). Snails in the control group had significantly more change in aperture width than snails in the crayfish treatment (*P* = 0.03).

Similarly, a significant treatment effect was found for aperture width (*F*_[1,30,0.05]_ = 6.94, *P* = 0.011; Fig. 4). Snails exposed to crayfish had significantly wider apertures than those within control trials. Significantly more change was observed in aperture width for control trials than was observed in crayfish trials (*F*_[1,30,0.05]_ = 4.95, *P* = 0.03; Fig. 4).

**Fig. 5:**
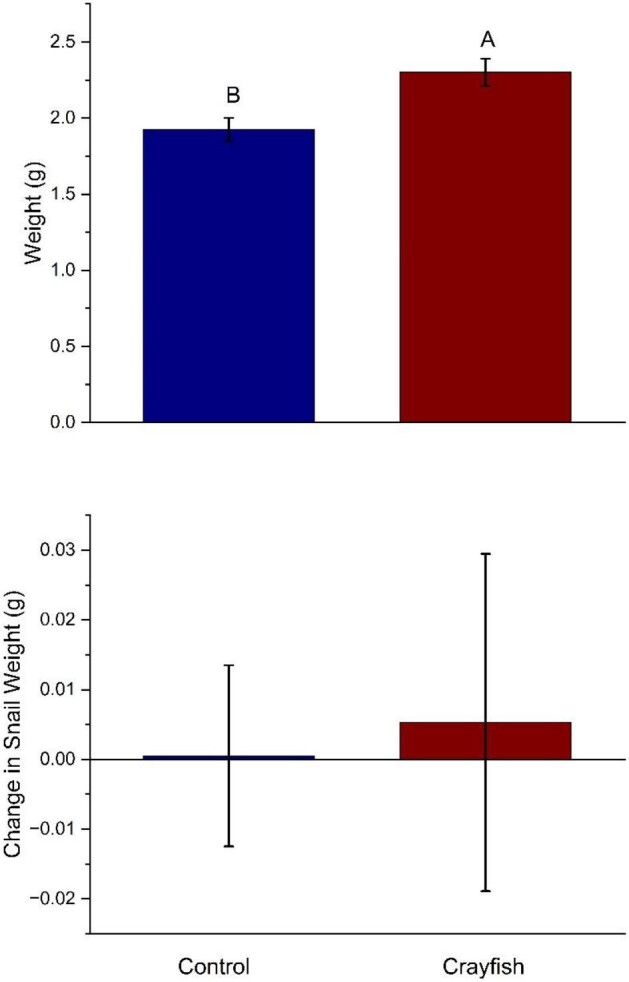
Total weight (top panel) and change in weight (bottom panel) within different treatments. The measurement is the mean (±SEM) for snails in the control (left bar) and crayfish (right bar) treatments. Within each panel, bars with different capital letters above the bar indicate a significant difference using an linear mixed model followed by a Tukey-HSD post hoc test. Snails in the crayfish treatment were significantly heavier than snails in the control treatment (*P* = 0.008). There were no significant differences observed for total change in weight when comparing crayfish trials to control trials (*P* = 0.88).

Next, the total weight of snails had significant treatment effects (*F*_[1,30,0.05]_ = 8.01, *P* = 0.008; Fig. 5). Specifically, snails in crayfish trials weighed significantly more than those in control trials. There were no significant differences observed for change in total weight (*F*_[1,30,0.05]_ = 0.02, *P* = 0.88; Fig. 5).

## Discussion

Predator presence altered snail morphology and antipredator tactics. Snails were never in direct contact with crayfish predators, indicating that morphological and physiological changes exhibited in snails were modulated by chemical cues. Our results are indicative that snails exposed to crayfish diverted energy from growing wider apertures to growing longer, stronger shells that increased the force necessary to crush their shells. Presumably, this morphological change provides protection from predators that rely on crushing shells to consume snails. Specifically, we found that greater force was needed to crush shells of snails that had been exposed to crayfish as opposed to snails in the control treatment that had no crayfish exposure throughout the experimental period, although this difference could simply be a function of differences in length (Fig. 2A, B). We also found that snails in the crayfish treatment had longer shells than the control treatment, and crayfish-exposed snails exhibited significantly more change in shell length than snails in the control (Fig. 3). Similarly, snails exposed to crayfish had wider apertures than the controls (Fig. 4). However, when measuring aperture change, our results showed that snails in the control treatment experienced significantly more change than snails in the crayfish treatment (Fig. 4). This points to snails altering resource allocation as a function of predator exposure, as snails in the control treatment placed more energy into aperture growth as opposed to snails in the crayfish treatment that put forth more effort to grow longer shells. These differences in change (i.e., change in length and aperture width) also lend evidence that predator exposure may alter the rate at which prey organisms grow as a predator avoidance tactic. Finally, snails in the crayfish treatment were heavier than control snails (Fig. 5). Combined, these results are indicative of a morphological response of snails to predator cues, where predator-exposed snails grew stronger and larger in order to be more successful in predator evasion, as crayfish tend to prey upon snails by crushing their shells before moving on to a “sucking” behavior in which crayfish remove the operculum and remove the tissue from inside the shell (Perry et al. 1997). Previous work has shown that longer and more rotund snails experience a reduction in mortality by crayfish predators because of the difficulty crayfish experience in handling and crushing these snails (Miranda et al. 2016). Overall, we found that crayfish exposure resulted in larger snail body size, which is similar to what is found within other studies (Alexander and Covich 1991). Thus, our study highlights the importance of the sensory cues of a predator's presence in mediating inducible defenses in prey.

Our current findings add further evidence that morphological changes in prey may decrease potential predation risk. Because chela crushing strength in crayfish is directly correlated to chela size (Benson and Clotfelter 2023), increased snail shell growth and shell strength in snails exposed to crayfish odors may provide either reduced predation threats or even predation refuges. Specifically, snails that grow larger may be better protected from crayfish predation because crayfish may not be able to fully clasp snails or crush shells with their chelae. This morphological response to predators illustrates the plasticity in potential responses from prey that can alter the likelihood of being consumed by predators (Brönmark and Miner 1992). In this way, the ability of prey organisms to adapt to risky situations in order to participate more efficiently in various activities (i.e., foraging, mating, caring for young, etc.) via inducible defenses is of great importance to prey individuals. Thus, morphological changes may be beneficial in increasing protection against predators in specific contexts.

Antipredator tactics are important components of predator–prey interactions, as these defenses may help to mitigate negative impacts on prey (Palmer and Packer 2021). The detection of predation risk within a habitat is not constant and varies along a continuum (i.e., low-risk to high-risk situations) due to spatial and temporal variations in predator movement. As such, the morphological responses that prey exhibit in response to risk should also exhibit plasticity based on the level of perceived risk. For instance, Belgrad et al. (2023) found that oysters (*Crassostrea virginica*) exposed to chemical cues from predatory blue crabs (*Callinectes sapidus*) formed thicker shells, and, in both laboratory and field studies, oysters with induced defenses had higher survivability than oysters not previously exposed to blue crabs. This is likely linked to increased energy required for mesopredators to break apart the shells and consume oysters (Belgrad et al. 2023). In this way, antipredator tactics in the form of changing morphology as a function of predator presence altered the outcome of the interactions between oysters and their predator. Also, similar to our own study, 12 populations of native marine snails (*Littorina obtusata*) increased in shell thickness when exposed to predatory green crab (*Carcinus maenas*) kairomones, which reduced snail vulnerability to being crushed by crabs (Corbett and Trussell 2024). Our study demonstrates that risk in habitats can directly influence the morphology and inducible defenses of prey, which fits into existing literature on this topic (see Adler and Harvell 1990; Auld and Relyea 2011; Hoverman and Relyea 2012). Because risk is dynamic and exists along a continuum, understanding the multitude of ways in which prey respond to risk can better inform researchers on the potential antipredator tactics prey may display as well as how these antipredator tactics may influence the outcome of interactions.

## Supplementary Material

obae033_Supplemental_File

## Data Availability

Data will be made public and available through the University of Michigan Biological Station data hub, Mfield, upon publication of the article.
