# Supplementary material for: Risky Business: Predator Chemical Cues Mediate Morphological Changes in Freshwater Snails
Source: Integr Org Biol. 2024 Sep 2;6(1):obae033. doi: 10.1093/iob/obae033 (PMC11404504; doi:10.1093/iob/obae033)
Supplement: obae033_Supplemental_File [file obae033_supplemental_file.docx]

**Supplemental Data**

**Table 1. Averages (± SEM) of all measures taken for each dependent variable across all treatments.**

| Dependent Variable | Treatment | Average ± SEM |
| --- | --- | --- |
| Total Crush Force | Natural | 141.35 ± 9.26 |
|  | Control | 126.61 ± 3.56 |
|  | Crayfish | 145.60 ± 4.34 |
| Crush Force per Length | Natural | 76.03 ± 3.86 |
|  | Control | 61.69 ± 1.63 |
|  | Crayfish | 66.98 ± 1.97 |
| Crush Force per Weight | Natural | 125.69 ± 11.22 |
|  | Control | 80.82 ± 3.31 |
|  | Crayfish | 77.67 ± 3.44 |
| Total Length | Natural | 1.84 ± 0.07 |
|  | Control | 2.07 ± 0.03 |
|  | Crayfish | 2.20 ± 0.03 |
| Change in Length | Control | -0.002 ± 0.004 |
|  | Crayfish | 0.02 ± 0.004 |
| Aperture Width | Natural | 1.03 ± 0.03 |
|  | Control | 1.16 ± 0.02 |
|  | Crayfish | 1.23 ± 0.02 |
| Change in Aperture Width | Control | 0.051 ± 0.006 |
|  | Crayfish | 0.028 ± 0.006 |
| Total Weight | Natural | 1.47 ± 0.12 |
|  | Control | 1.93 ± 0.08 |
|  | Crayfish | 2.30 ± 0.09 |
| Change in Weight | Control | 0.005 ± 0.02 |
|  | Crayfish | 0.0005 ± 0.01 |

**Figure S1.** This figure shows violin dot plots of crush force data, with crayfish exposed snails represented in red and control snails represented in green. The first panel shows the total crush force (N) of each snail in both treatment groups. The middle panel shows the total crush force per length (N/cm) of snails in both treatment groups. Finally, the last panel shows the total crush force per weight (N/g) of all snails in both treatment groups.


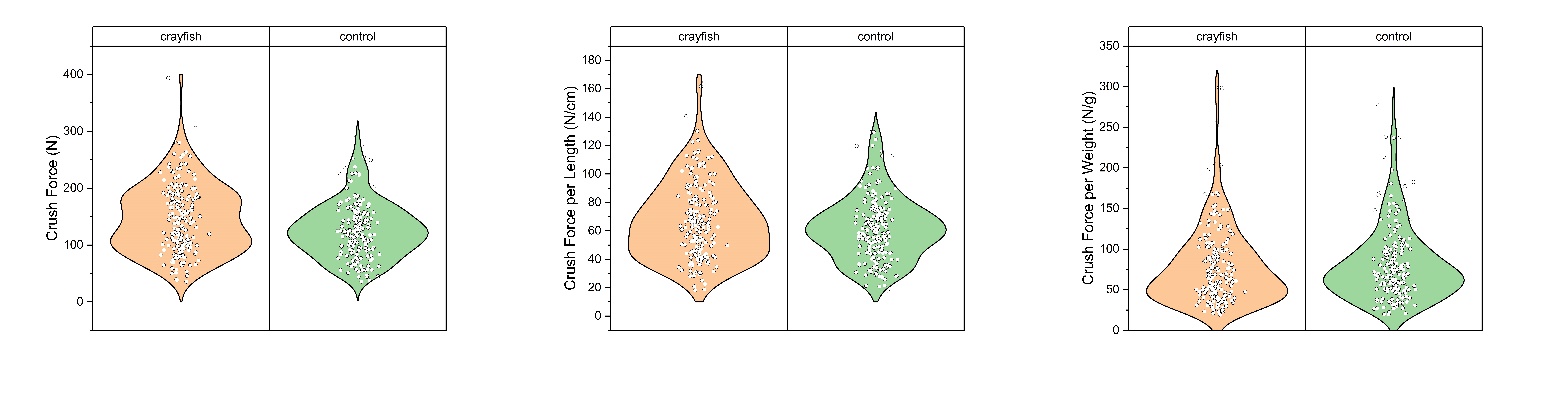


**Figure S2.** This figure shows violin dot plots of changes data, with crayfish exposed snails represented in red and control snails represented in green. The first panel shows total change in aperture width (cm) for all snails in both treatment groups. The middle panel shows total change in shell length (cm) for all snails in both treatment groups. Finally, the last panel shows total change in weight (g) of all snails in both treatment groups.

**
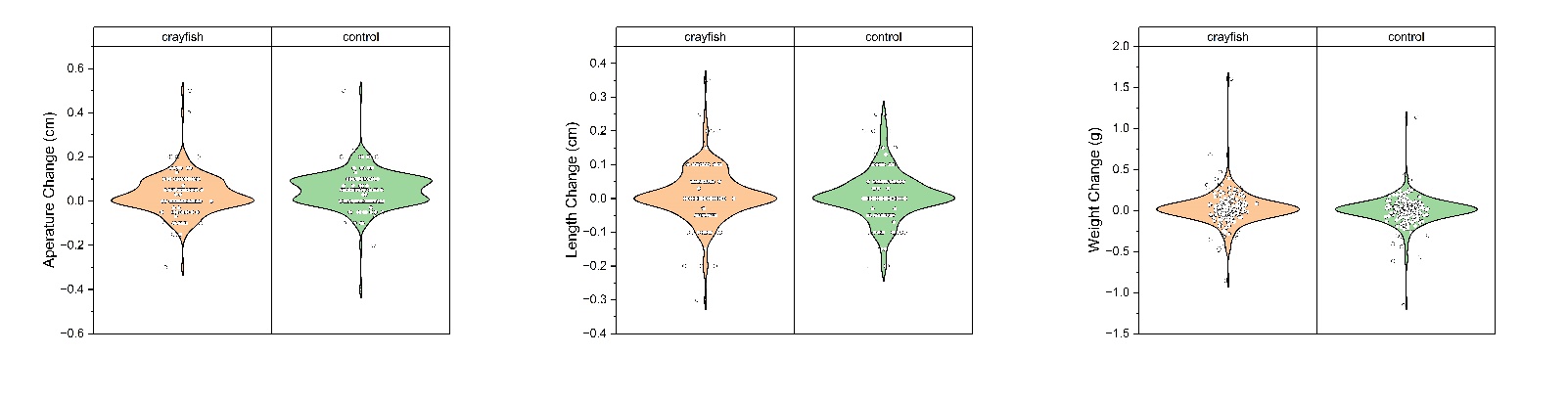
**

**Figure S3.** This figure shows violin dot plots of all final measurement data, with crayfish exposed snails represented in red and control snails represented in green. The first panel shows the total aperture width (cm) of all individuals in both treatment groups. The middle panel shows the total shell length (cm) of all individuals in both treatment groups. Finally, the last panel shows the total weight (g) of all individuals in both treatment groups.

**
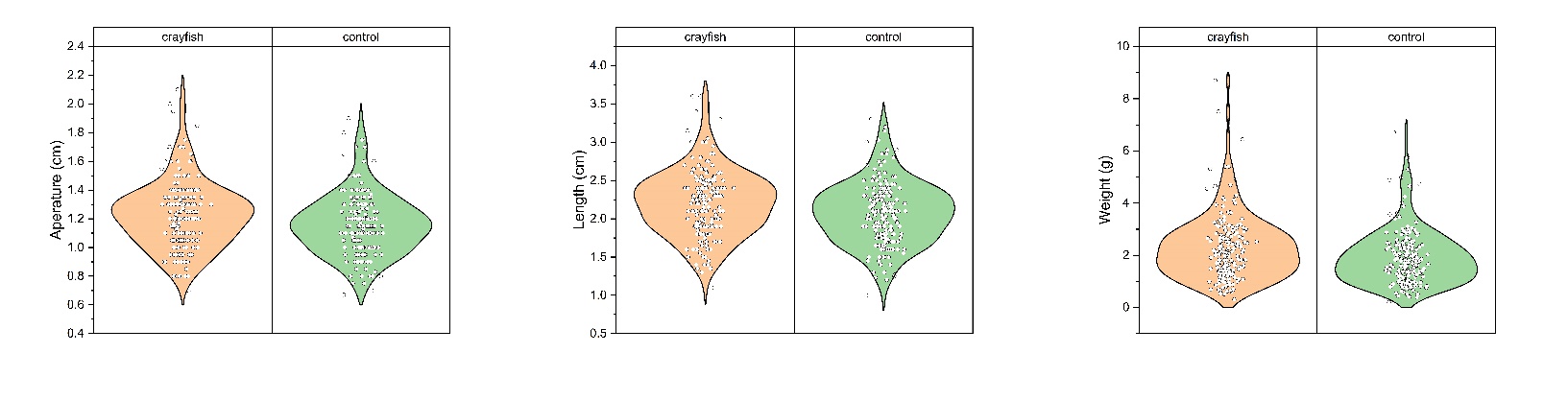
**
